# The Atlanta Obstetrical Society

**Published:** 1894-08

**Authors:** 


### THE ATLANTA OBSTETRICAL SOCIETY.

In June, 1893, in the office of Drs. Kime & Davis there assembled six physicians: Drs. V. O. Hardon, Geo. H. Noble, J. C. Avary, W. A. Crow, R. R. Kime and E. C. Davis, for the organization of a society with the special purpose to study more carefully and reciprocally gynecology and obstetrics. This lead to the organization of the Atlanta Obstetrical Society which has just passed through a very successful year, under the management of the following officers: Dr. W. A. Crow, President; Dr. R. R. Kime, Vice-President; Dr. E. C. Davis, Secretary and Treasurer. One meeting was to be held each month, at which a paper was to be offered by one of the members. During the first year of the Society's existence the following papers have been read: The Management of Abortions, by Dr. G. H. Noble; Indications for the Induction of Artificial Abortions, by Dr. V. O. Hardon; Classification of Puerperal Infection, with Notes on Treatment, by Dr. R. R. Kime; Chancroids of the Female Generative Organs, by Dr. E. C. Davis; A Case of Chronic Cystitis and Urethritis with Ulcers, by Dr. C. D. Hurt; Tampons, by Dr. J. C. Avary; Vomiting of Pregnancy, by Dr. J. G. Earnest; Nausea, by Dr. J. B. Baird. Interesting cases have also been reported at each meeting.

The membership has been increased to thirteen. The initiation fee, one dollar, exacted from each member, has proven sufficient to defray the expenses of the organization.

Altogether the first year of the Society has been a very successful one, and it enters upon the second with very encouraging auspices. The officers now are: Dr. J. B. Baird, President; Dr. C. D. Hurt, Vice-President; and Dr. E. C. Davis, Secretary and Treasurer.

---

*The editor of this Journal is engaged in collecting data relative to the History of Medicine and Surgery in Georgia, to be published in these pages at the proper time. Our State has furnished many distinguished names to the roll of American physicians and surgeons, and it is eminently proper that some systematic and enduring record be made of their work.*

*If any of our readers can give us any information which will be of value in this connection we will esteem it a great courtesy. Kindly address the* MANAGING EDITOR.

